# An assessment of various blood collection and transfer methods used for malaria rapid diagnostic tests

**DOI:** 10.1186/1475-2875-6-149

**Published:** 2007-11-15

**Authors:** Jennifer Luchavez, Ma Eichelle Lintag, Mary Coll-Black, Fred Baik, David Bell

**Affiliations:** 1 Research Institute for Tropical Medicine, Filinvest Compound, Alabang, Muntinlupa, Philippines; 2 World Health Organization – Regional Office for the Western Pacific, UN Avenue, Manila, Philippines

## Abstract

**Background:**

Four blood collection and transfer devices commonly used for malaria rapid diagnostic tests (RDTs) were assessed for their consistency, accuracy and ease of use in the hands of laboratory technicians and village health workers.

**Methods:**

Laboratory technicians and village health workers collected blood from a finger prick using each device in random order, and deposited the blood either on filter paper or into a suitable casette-type RDT. Consistency and accuracy of volume delivered was determined by comparing the measurements of the resulting blood spots/heights with the measurements of laboratory-prepared pipetted standard volumes. The effect of varying blood volumes on RDT sensitivity and ease of use was also observed.

**Results:**

There was high variability in blood volume collected by the devices, with the straw and the loop, the most preferred devices, usually transferring volumes greater than intended, while the glass capillary tube and the plastic pipette transferring less volume than intended or none at all. Varying the blood volume delivered to RDTs indicated that this variation is critical to RDT sensitivity only when the transferred volume is very low.

**Conclusion:**

None of the blood transfer devices assessed performed consistently well. Adequate training on their use is clearly necessary, with more development efforts for improved designs to be used by remote health workers, in mind.

## Background

Malaria rapid diagnostic tests (RDTs) are an increasingly important aspect of malaria case management in remote rural settings where good microscopy is difficult to maintain [1]. In these settings, RDTs are often used by health workers with limited training or supervision. The success of their introduction depends on their accuracy, which is in part dependent on end-users to properly and consistently perform the tests. RDTs are lateral flow tests detecting malaria parasite antigen in whole blood to produce a visible line on a nitrocellulose wick. Blood volume and, therefore, antigen load, is expected to affect test sensitivity, while excess blood volume may cause background staining and obscure weak test lines. Simplicity and accuracy of blood collection from finger-prick and transfer to the RDT may thus be crucial to overall test performance. As RDTs are likely to be used in poorly regulated environments, blood safety is also a major concern.

Poor preparation of RDTs by users is well documented [2-6]. However, there is surprising variety of methods available in commercially available tests to perform the simple function of transferring blood from a finger prick to the device. As most RDTs are essentially similar in design, it would seem that certain transfer devices must be better suited to this task than others.

Most RDTs are produced either as cassettes, in which a blood sample is placed directly onto an encased nitrocellulose wick, or as dipsticks, in which blood from the finger prick or in a well is directly applied to the wick. This paper reports an assessment of the consistency, accuracy and ease of use of blood transfer techniques designed for cassettes and dipsticks, and the effects of variable blood volume on RDT results.

## Methods

### Study population and devices

**Figure 1:**
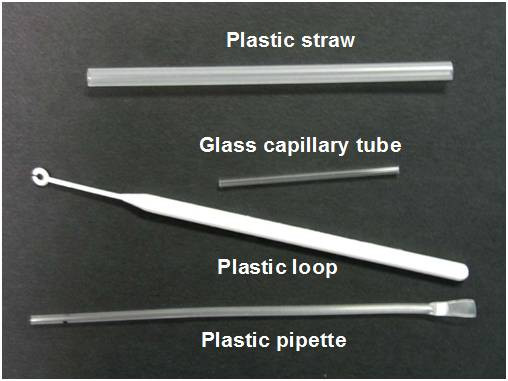
Blood collection and transfer devices. The plastic straw and plastic loop collect a film of blood by touching the finger prick; the glass tube collects blood by capillary action, while the plastic pipette aspirates blood similar to a conventional dropper.

This study was performed in the Philippines, using four commonly used blood transfer devices: a plastic straw and a plastic loop (both from Paracheck *Pf*, Orchid Biomedicals, India), a plastic pipette (OptiMal-IT, Diamed AG, Switzerland), and glass capillary tube (ICT Malaria Combo Cassette, R&R Marketing, South Africa) (Figure 1). The straw, loop and capillary tube are designed to collect 5 μL by touching the blood drop on the finger prick, while the plastic pipette is intended to aspirate 10 μL. Except for the pipette, which is designed to transfer blood into a well, these devices are designed for use with cassette-type RDTs. Only adults were used as donors for blood sampling.

Consistency of transferred blood volume by straw, loop and pipette was assessed in the field by 20 tertiary-trained laboratory technicians (mean age = 30) and 36 village health workers (VHWs: mean age = 35). VHWs had either primary or secondary education and had been working as VHWs for more than one year. All had previous experience in blood collection and malaria blood film preparation. Sixty-six percent (37/56) had prior experience with straw and/or loop transfer devices for RDTs. The glass capillary tube was assessed in the field by a separate group comprising 30 laboratory technicians (mean age = 36) and 18 VHWs (mean age = 45), of whom 60% (29/48) had previous experience with the straw and/or loop. Assessment of the capillary tube was done differently from the other devices as described in the next section, hence a separate group was involved in the study.

### Consistency and accuracy of blood volume transferred

The average volume transferred by each device under ideal laboratory conditions was measured by technicians familiar with all devices. Volume estimation was complicated by the varying device designs. To assess the straw and plastic pipette, blood samples were serially taken up with the use of a new device each time from a microtube containing 100 μL of blood and deposited onto filter paper (Whatman 3 M). The volume transferred by the loop was estimated by collecting blood from a 100 μL drop on parafilm to simulate blood collection from a finger prick in a horizontal position. Straw and pipette samples were collected from a microtube, as this was functionally similar to a large drop on a finger. The number of samples drawn by each device was tallied and divided by the volume of blood used. Because the capillary tube did not readily release blood onto the filter paper, a different method was used to estimate the volume transferred by the capillary tube. Samples of 5 μL were transferred into the glass capillary tube using a calibrated micropipette and subsequently deposited into its associated RDT cassette. Volume was estimated by measuring the initial and residual volume in each capillary tube (height of column).

In the field, each health worker was briefly instructed verbally on correct use of each device before performing a finger prick with the use of a standard lancet. Blood was collected from the same finger prick using each device in random order. After the blood spot had dried on the filter paper, the diameter was measured and the mean area calculated. For the capillary tube, volume delivered was estimated by subtracting the residual from the initial height of column, and the mean calculated. These results were compared with the areas or height of accurately measured volumes of 5 and 10 μL blood from a micropipette.

Significance was calculated using Student's T-test and Chi-square test (Stata Version 6 – StataCorp LP, USA).

### Ease of use

Using each device, health workers were requested to transfer blood from a single finger prick to a cassette RDT (ICT Malaria Combo Cassette, R&R Marketing, South Africa) previously determined to have a blood window suitable for all devices. The devices were presented to each health worker in alternating order. Ease of use was determined by observation based on three criteria: (1) obtaining blood in device in accordance with manufacturer's instructions, (2) transferring blood without loss, and (3) releasing blood onto the RDT cassette, judged by a clear blood stain on the cassette pad and absence of an intact blood film in the case of the loop and straw. Successful transfer of blood was based on transfer on (1) initial attempt, and (2) within one minute of commencing attempt. An observation checklist and a simple questionnaire assessed the health workers' preferred device and the reason for their preference.

### Effect of blood volume on RDT sensitivity

The effect of varying blood volume on RDT sensitivity was tested by applying serial volumes (1–20 μL) of pre-prepared cryo-preserved *Plasmodium falciparum *parasitized blood diluted to 200 and 2,000 parasites/μL [7] with a micropipette. RDTs were otherwise performed according to the manufacturer's instructions. The intensity of the *P. falciparum*-specific test band was assessed blinded by three trained laboratory technicians from 0 (no band) to 3 (strongest band) using a standardized RDT rating chart.

### Ethical considerations

The study was approved by the Institutional and Ethical Review Board of the RITM, Department of Health, Republic of the Philippines. All participating blood donors and health workers gave written informed consent.

## Results

Under ideal laboratory conditions, all four devices consistently delivered the intended volume of blood. The straw, loop and plastic pipette delivered a mean of 5.56 (sd = 0.98), 5.26 (sd = 0.64) and 10.53 μL (sd = 0.88) of blood respectively onto the filter paper while the glass capillary tube transferred a mean 4.81 μL (sd = 0.92).

In the field, there was high variability in volumes delivered by the different techniques, with all four devices transferring significantly different volumes than those intended. The straw and the loop both transferred mean volumes greater than intended, while the glass capillary tube and the plastic pipette transferred less than the intended volume (Table 1).

**Table 1:** Comparison of mean area of blood spots or mean height of blood collected by various techniques against pipetted standard volumes in controlled laboratory conditions.

Device	Mean area (mm^2^) or height (mm) of blood collected	Sd	Mean area (mm^2^) or height (mm) of pipetted standard blood volumes (n = 20)	sd	P value^a ^(one-tailed)
Straw (n = 56)	27.50	18.51	14.69	3.32	P < 0.05
Loop (n = 56)	25.27	10.74			P < 0.05
Plastic Pipette (n = 56)	14.66	12.97	25.84	6.01	P < 0.05
Glass Capillary Tube^b ^(n = 44)	3.35^c^	1.76	4.75^c^	0.34	P < 0.05

^a ^Difference between actual and intended (pipetted) volumes.

^b ^Different set of health workers used. Four further technicians failed to deliver any blood, and results are not included.

^c ^mean height

sd standard deviation

No device performed consistently well when used by all groups of health workers. Although success in blood transfer tended to be higher with the glass capillary tube and straw, differences in successful transfer (some blood transferred versus none or failure to transfer any) were not statistically significant between any device (P > 0.06). Overall, previous experience with RDTs was a significant positive factor in preventing failed blood transfer (p = 0.02).

Many health workers were unable to aspirate the intended volume with the plastic pipette (with a soft tube but does not have a balloon to squeeze). No participant was successful in filling the pipette to the required 10 μL mark. Although 41 out of 46 (89%) participants were successful in delivering some volume of blood to the RDT with the glass capillary tube (Table 2), 15 of them (33%) delivered less than 3 μL. Five (11%) were unable to deliver any blood at all due to loss of the capillary effect through intrusion of air below the blood in the tube.

**Table 2:** Successful collection and transfer of blood to RDT using various devices.

Device	Health workers' previous experience in RDT use^a^	n^b^	Successful collection and transfer of blood to RDT		Failed collection or transfer of blood to RDT	p-value (failure rate between experienced vs. non-experienced
					
			on initial attempt	w/in 1 minute or on repeat attempt		
Straw	No	16	9	6	1	0.589
	Yes	29	23	5	1	
Loop	No	17	5	7	5	0.054
	Yes	29	17	10	2	
Plastic	No	15	3	10	2	0.111
Pipette	Yes	29	10	19	0	
Glass	No	17	13	2	2	0.619
Capillary Tube	Yes	29	24	2	3	

^a ^Health workers with experience in using RDTs with the straw and/or loop.

^b ^Some health workers were not able to use devices as insufficient blood was obtained from the fingerprick.

The health workers cited the straw as the most preferred device (15/48 or 31%), followed by the loop (11/48 or 23%), and the glass capillary tube (8/48 or 17%), while the least preferred was the plastic pipette (6/48 or 12%).

**Figure 2:**
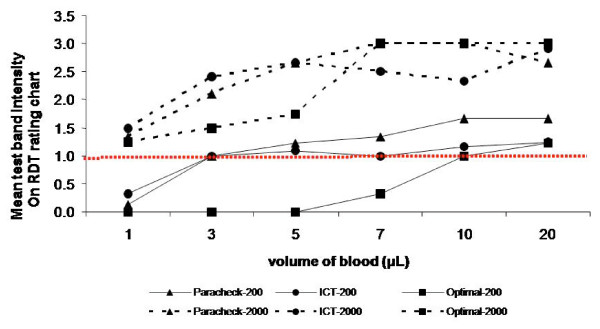
Test band intensity of various RDTs using different blood volume and parasitaemia. At 200 parasites/μL blood, using less blood than the required volume did not give clearly visible test bands (less than 1 on the RDT rating chart). At 2000 parasites/μL blood, all RDTs gave visible test bands (1 or higher) in all the blood volumes used. Note that Optimal is designed to use 10 μL of blood, the other tests 5 μL.

Inadequate blood volume reduced sensitivity, with false negative results occurring with low parasite density samples (200 p/μL), when volumes less than 3 μL (for Paracheck and ICT) or 10 μL (Optimal) were used (Figure 2). Excess blood volume up to 20 μL did not affect the test results significantly, although background staining, which can obscure faint results, was common.

## Discussion

This study demonstrated a great deal of variation in consistency and accuracy of volume delivered by four commercially-available blood transfer devices. However, the RDTs with which they are marketed all performed well over a wide range of volumes (up to 20 μL) at the low and medium parasite densities, suggesting that excess blood volume and volume consistency are not crucial to test performance in the field. As expected, insufficient blood volume is critical, causing false-negatives results at low parasite density at volumes about 30% below those intended. Thus, emphasis should be placed on ensuring the delivery of at least adequate blood volumes rather than stressing avoidance of overloading RDTs.

Among the four devices, the straw appears to be the device of choice for its ease of use and consistently adequate delivery of blood volume. However, its wide bore poses difficulty when drawing blood from children. While the loop also delivers adequate blood volumes when transfer was successful, it was frequently unsuccessful in transferring any despite previous experience of the technicians. Several participants expressed strong dislike of the loop and commented that in practice they use the handle rather than the actual loop to collect and transfer blood, or alternatively, deposit blood directly from the finger onto the RDT. Similar experience is reported elsewhere [7]. The capillary tube and pipette both had problems through insufficient delivery of blood volume. In the case of the pipette at least, this should be relatively simple to correct through re-design to include a compressible bulb to increase the blood volume that can be aspirated, and other commercially-available devices include this.

In addition to delivery of adequate blood volume and ease of use, blood safety remains an important aspect when assessing blood transfer devices. Though blood safety is not qualitatively measured in this study, blood flicking from the loop, observed a number of times during the study and noted elsewhere [8], and the potential for breakage of the glass capillary tube, may be reasons for caution.

## Conclusion

Blood collection and transfer would appear an uncomplicated and simple activity, yet the designs of existing devices render them more difficult to manipulate than expected. Obtaining a large blood drop from the initial finger prick will minimize problems with all devices, and this can be difficult with children in particular. An ideal device should be simple to use and deliver at least a minimum volume of blood, while maintaining blood safety. While many resources go into the development of the actual test devices, this study suggests more development effort on simple blood transfer devices may deliver significant benefits to diagnostic performance.

## Authors' contributions

JL coordinated the study both in the laboratory and in the field and contributed in its conception and design, analysis and interpretation of data, and wrote and revised the manuscript. MEL and MCB carried out the field data collection and helped in the analysis and interpretation. FB performed statistical analysis and edited the manuscript. DB identified problems associated with RDTs, conceptualized and designed the study, and critically reviewed the manuscript. All authors read and approved the final manuscript.
